# Regulation-based probabilistic substance quality index and automated geo-spatial modeling for water quality assessment

**DOI:** 10.1038/s41598-021-02564-w

**Published:** 2021-12-10

**Authors:** Artyom Nikitin, Polina Tregubova, Dmitrii Shadrin, Sergey Matveev, Ivan Oseledets, Maria Pukalchik

**Affiliations:** 1 Center for Computational and Data-Intensive Science and Engineering, Skolkovo Institute of Science and Technology, Moscow, Russian Federation 121205; 2 Digital Agriculture Laboratory, Skolkovo Institute of Science and Technology, Moscow, Russian Federation 121205; 3 Faculty of Computational Mathematics and Cybernetics, Lomonosov Moscow State University, Moscow, Russian Federation 119991; 4 Marchuk Institute of Numerical Mathematics of Russian Academy of Science, Moscow, Russian Federation 119333

**Keywords:** Environmental sciences, Applied mathematics

## Abstract

Natural environments are recognized as complex heterogeneous structures thus requiring numerous multi-scale observations to yield a comprehensive description. To monitor the current state and identify negative impacts of human activity, fast and precise instruments are in urgent need. This work provides an automated approach to the assessment of spatial variability of water quality using guideline values on the example of 1526 water samples comprising 21 parameters at 448 unique locations across the New Moscow region (Russia). We apply multi-task Gaussian process regression (GPR) to model the measured water properties across the territory, considering not only the spatial but inter-parameter correlations. GPR is enhanced with a Spectral Mixture Kernel to facilitate a hyper-parameter selection and optimization. We use a 5-fold cross-validation scheme along with $R^2$-score to validate the results and select the best model for simultaneous prediction of water properties across the area. Finally, we develop a novel Probabilistic Substance Quality Index (PSQI) that combines probabilistic model predictions with the regulatory standards on the example of the epidemiological rules and hygienic regulations established in Russia. Moreover, we provide an interactive map of experimental results at 100 m^2^ resolution. The proposed approach contributes significantly to the development of flexible tools in environment quality monitoring, being scalable to different standard systems, number of observation points, and region of interest. It has a strong potential for adaption to environmental and policy changes and non-unified assessment conditions, and may be integrated into support-decision systems for the rapid estimation of water quality spatial distribution.

## Introduction

Freshwater—probably the most precious resource on the planet—plays a crucial role for humans since it is exploited in farming, industry, domestic consumption, and power supply^1–4^. Yet, in the light of drastically changing environmental conditions freshwater resources are highly vulnerable. They are affected both by natural climatic shifts as well as by anthropogenic impact manifested in pollution and catchment disturbance. To enhance freshwater storage protection active monitoring and quality assessment are required.

A freshwater quality assessment is complicated at both spatial and temporal scales and in terms of data collection. In other words, numerous points of observation are needed, some flows are partly hidden or even unavailable for the observers without specific equipment^5^. Another bottleneck for assessment is the high complexity and heterogeneity of water composition. It consists of a number of parameters of distinctive nature-physicochemical (such as acidity, alkalinity, turbidity, the content of cations and anions, including toxic chemicals, such as pesticides and toxic trace metals) and biological (presence and structure of living organisms’ community). These characteristics are highly interconnected with each other and sensitive to the external stressors and processes at the same time. Multiple factors, ranging from natural to anthropogenic ones, determine the significant spatial variability of water characteristics and the overall quality on large territories. The former includes aquifer characteristics heterogeneity, substance migration patterns, while the latter—diverse sources of potential pollution from different land-use types in urbanised and developed lands^6–8^.

In order to monitor complex natural systems, such as freshwater reservoirs, an investigator has to answer two key questions: (1) how to contemplate as much information as possible in a most conscientious way and (2) how to cover maximal territory using the available data, which tends to be quite limited. A wide-spread approach to tackle the first problem is to evaluate the water system state by reducing the overall complexity, e.g. by calculating one integrative parameter, such as Water Quality Index (WQI). The idea of introducing a single aggregated parameter, such as WQI, was firstly proposed by Horton, 1965^9^. It has been significantly elaborated since then^10–13^, being used even by some governmental agencies, such as National Sanitation Foundation Water Quality Index (NSFWQI), Canadian Council of Ministers of the Environment Water Quality Index (CCMEWQI), British Columbia Water Quality Index (BCWQI)^14,15^. The quality index approach is widespread in assessing other complex natural environments, e.g. soil^16–18^. The main objective of classic WQI is the aggregation of multiscale data, based on the relative importance of parameters, and further categorisation according to the obtained results. However, the applicability of WQI raised a number of concerns as it lacks unity and coherence in estimation workflow and evaluating the parameters.

As a consequence, these factors led to a high divergence in interpreting the obtained results^15,19–22^. The existing aggregation outlooks rarely reflect the normative thresholds directly^23^, and often miss other than “less is better” possible motivations for parameters’ consideration. Thus, the cases of the optimum range, when the permissible parameter content is defined by some lower and upper bounds, are underrepresented. Significant part of the recent developments focuses on to the approaches of picking up the most important features to construct the index from them via assigning different weights^24–26^ in the contrast to subjective recommendations^27^, or systematizing them, using such tools as Multi-Criteria Decision Analysis, Analytic Hierarchy Process, Fuzzy Logic^10,14,28^. In case of implementing the expert opinion systematization techniques the authors identified several significant uncertainties accompanying a non-stable data aggregation process and a high risk of misinterpretation. Some of the new approaches are based on implementing numerical tools to consider the overall variability of characteristics across the territory of study, e.g. use of the Principal Component Analysis (PCA). However, if the high variance reflects noticeable parameter changes from an excellent to an appalling state, low variance does not allow to distinguish whether conditions are very poor or not. Finally, one may doubt whether it is expedient to aggregate the information into one index value at all after measuring tens of parameters. Specifically, considering that monitoring observations and private assessments are already based on the plethora of different characteristics^29^. Thus, the development of the new unified (i.e. non-specific to study sites) approaches to quality assessment are needed^30,31^.

In terms of observation and monitoring water quality spatial dynamics, data imputation, and prediction of possible system shifts, modeling approaches are in common use. Among them, two modeling approaches can be distinguished: process-based (PB) and data-driven solutions. Classic PB solutions are widely used for the tasks such as description of transport and fate of contaminants in water flows^32^, recharge-depletion and consumption dynamics^33^. These approaches, built on structural equations, are connected with the description of stochastic processes underlying visible outputs with specified initial and boundary conditions^34^. Despite being comprehensive and fundamental, i.e. based on the observed dependencies in exploratory researches, the PB solutions are often considered as too complicated. Such models are usually limited by the demand of complex explanatory infrastructure related to various natural environments to describe the principles behind the processes; up-scaling challenges, slow and clumsy calculations, as well as biases caused by the established assumptions and conditions^34–36^.

A suitable solution in the environmental modeling and assessment is using the data-driven modeling solutions, to be more specific, machine learning (ML) to supply and improve PB techniques and as a self-contained approach. In the last few decades the popularity of ML-based approaches used for the modeling the water characteristics’ distribution, including over-all water quality, has been increasing. The ML techniques have been successfully introduced in the evaluation of the most important aspects of freshwater reservoirs, e.g. surface water quality and its mapping^37^, determining the key parameters for accurate quality estimation^38^, predicting groundwater contamination^39,40^ and level dynamics^41^. Although ML approaches require relatively large training sets and leave behind the physical mechanisms of processes, combined with geostatistical techniques, they allow to establish the distribution of characteristics more precisely and in higher resolution on both spatial and temporal scales. As compared to the PB modeling techniques, the ML approaches allow to model complex non-linear relationships between independent and target parameters using black-box approaches^42,43^. Thus, there is no need to rely on any empirical models that are not always able to embrace all aspects of the considered system. This in turn opens an avenue for geo-spatial modeling automatization.

One of the most popular tools for successful predicton of the spatial distribution of parameters related to the natural environment (e.g. water quality, groundwater level, soil organic matter, air pollutant) is the implementation of Gaussian Process (GP)^44–47^. GP is a kernel-based model able to handle different types of input data without any limitations of the particular parametric form of relation to the output. The flexibility of GP allows to use it for the most frequent tasks in environmental studies such as regression (also called kriging) or classification^48^ with the ability to perform simultaneous multi-parameter predictions giving confidence for the predicted values. Apart from the advantages of GP, a list of weaknesses is normally mentioned: limited computational efficiency with the growing number of samples, poor scaling with increasing data dimensionality. Additionally, it requires to choose variogram and mean (trend) functions structure and make some assumptions about the data distribution type (e.g., normality)^49^. To handle the efficiency problems several approaches may be exploited, including batch-learning^50^, kernel approximation^51^ and dimensionality reduction techniques^52^. Therefore, considering a high popularity of GP in the environmental science community, the studies applying GP to environmental issues and showing the ways to decrease handcrafting (i.e., through the automated kernel structure selection) and increase computational efficiency are of paramount interest and practical importance. It should be noted, that there are other ML tools potentially capable of obtaining similar results, e.g. Support Vector Regression^53^, neural networks^54–56^.These models may give reasonable results, however black-box approaches are usually reported to be difficult in interpretation. At the same time, GP benefits over the above mentioned approaches due to the results of GP applications are usually easier interpreted. Still, the comparison between different modeling frameworks to solve the multi-task problems might be the promising direction in advancing assessment approaches.

This paper presents a part of the project aimed to implement the ML techniques to the environmental monitoring issues. Considering the above-mentioned developments of the community, the objective of this research is to show an automated Geographical Information Systems (GIS) approach for the freshwater assessment and spatial modeling applied to the existing sample network based on the data including 1526 samples obtained from 448 unique points across the New Moscow region described by 21 parameters and spatial coordinates^57^.

The detected concentrations of parameters used in the modeling vary significantly across the territory. Some of them, e.g. Cl, NO$_3$, PO$_4$ ions, as well as metal ions, Fe, Mn, Ni, may exceed established permissible limits 2-8 times while their content in other locations may be equal to 0. Our proposed modeling workflow is based on the multi-task Gaussian process regression (GPR) featured by the automatic kernel structure selection and hyper-parameter optimization. An important advantage of the developed approach is that it enables predicting the spatial distribution of all of the measured properties in one consistent procedure. Particularly, it considers both spatial and inter-parameter dependencies and allows to assess not only the precise values of water properties but also their probabilistic ranges as well as enables the accuracy control with the minimal user efforts. This modeling framework is supplied with a limit-driven assessment system: one can easily check whether the selected parameter is in the permissible range (considering the diapason, not only the upper limit) in the selected location. Considering the convenience of the joint concise characteristic to describe the overall system state, we propose a probabilistic substance quality index (PSQI). It incorporates both observations and the established regulations, denoting the probability that all of the characteristics lie in permissible diapasons. The presented approach is devised to meet the requirements to enhance reproducibility and fairness of the assessment. It is open to scaling to different standard systems, set of points of observation, region of interest and has a strong potential for adaption to environmental and policy changes and non-unified conditions of assessment. Therefore, it ensures direct integration into support-decision systems.

## Modeling tools and water quality index

**Figure 1:**
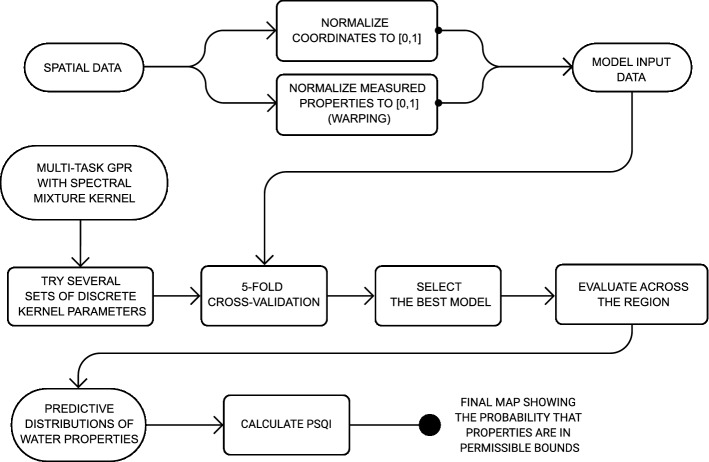
Main steps of the workflow: from data pre-processing to final Probabilistic Substance Quality index mapping.

In the following section the key concepts and stages of the proposed assessment approach are described in detail. Firstly, the theory behind the Gaussian process regression is explained and the modeling objectives are formulated. In order to deal with one of the key difficulties of the natural systems quality assessment—representative consideration of the overall complexity in spatial modeling—the concept of multi-task Gaussian process regression is given. It is supplied with an explanation of the automatization procedure (hyper-parameter selection) and details for reproducibility: data pre-processing, model validation, and technical requirements to handle calculations. Finally, the definition of the proposed Probabilistic Substance Quality Index is given. The general scheme of the workflow is presented in Fig. 1.

### Gaussian process regression

In order to perform geo-spatial modeling of multiple water properties from the collected dataset, we refer to the *Gaussian process regression* (GPR) framework^49^, known as *kriging* in geostatistics. *Mean* $\mu (\cdot )$ and *covariance* (or *kernel*) $k(\cdot , \cdot )$ functions completely determine a Gaussian process:

1 $$\begin{aligned} \begin{aligned} f(\mathbf {x})&\sim {\mathscr {G}}{\mathscr {P}}(\mu (\mathbf {x}), k(\mathbf {x}, \mathbf {x}^\prime )),\\ \mu (\mathbf {x})&= {\mathbb {E}}~f(\mathbf {x}),\\ k^x(\mathbf {x},\mathbf {x}^\prime )&= {\mathbb {E}}~[(f(\mathbf {x}) - \mu (\mathbf {x}))(f(\mathbf {x}^\prime ) - \mu (\mathbf {x}^\prime ))], \end{aligned} \end{aligned}$$

where ${\mathbb {E}}$ is a mathematical expectation and $\mathbf {x}\in {\mathbb {R}}^d$ is a vector of *d* input parameters, which are 2D coordinates in our case (for instance, represented in the Mercator projection). As an example, consider a simple GP model:

2 $$\begin{aligned} y(\mathbf {x}) = f(\mathbf {x}) + \epsilon , \end{aligned}$$

where $\epsilon \sim {\mathscr {N}}(0, \sigma ^2)$ accounts for noise in measurements, hence, helping to avoid model over-fitting. Given the training samples $\mathbf {X}=\left( \mathbf {x}_1,\dots ,\mathbf {x}_N\right) ^\intercal \in {\mathbb {R}}^{N \times d}$, ${\mathbf {Y}}=\left( y_1,\dots ,y_N\right) ^\intercal \in {\mathbb {R}}^N$, where *N* denotes the number of available measurements and $(\cdot )^\intercal$ denotes a transpose, the predictive distribution at arbitrary point $\mathbf {x}_*$ can be found as

3 $$\begin{aligned}&{\hat{f}}(\mathbf {x}_*) \sim {\mathscr {N}}({\hat{\mu }}, {\hat{\sigma }}^2),\\ {\hat{\mu }}(\mathbf {x}_*)&= \mu (\mathbf {x}_*) + k^x_*\Sigma (\mathbf {y}- \mu (\mathbf {X})),\\ {\hat{\sigma }}^2(\mathbf {x}_*)&= k(\mathbf {x}_*, \mathbf {x}_*) - (k^x_*)^T\Sigma ^{-1}k^x_*,\\ \Sigma&= K^x + \sigma ^2I, \end{aligned}$$

where $K^x = k^x(\mathbf {X}, \mathbf {X}) = k(\mathbf {x}_i, \mathbf {x}_j), i,j=1,\dots ,N$ and $k_*^x = k^x(\mathbf {X}, \mathbf {x}_*)$ are spatial covariance matrices between all of the training points and between training points and the single prediction point, respectively; $\mu (\mathbf {X}) = \mu (\mathbf {x}_i), i=1,\dots ,N$ is the mean vector-function evaluated at the training points; and *I* is an identity matrix. A choice of the mean and kernel functions depends on the assumptions about the model and the particular application. An example of a kernel function is a widely used *Gaussian* kernel, which corresponds to Gaussian variogram in kriging. The kernel hyper-parameters are usually optimized using *Maximum Likelihood Estimation* (MLE)^58^ or its variations.

**Figure 2:**
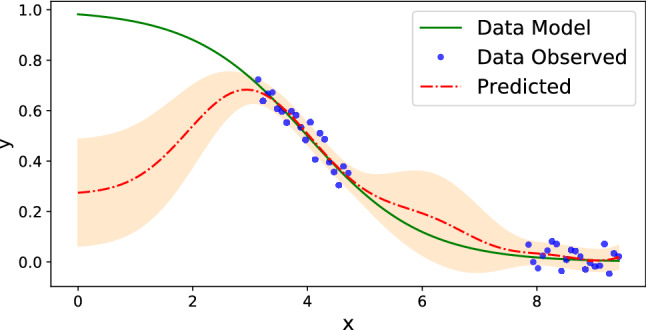
Example of Gaussian process regression (red dashed line stands for the predictive mean and orange fill stands for the standard deviation intervals) with noisy measurements (blue dots) of a sigmoid function (solid green line) using Gaussian kernel and constant mean function.

Figure 2 illustrates an example of GPR using the Gaussian kernel and the constant mean over the sigmoid function with noisy measurements. Predictive variance increases notably at the points with missing measurements. Moreover, outside of the interpolation region, a predictive mean fails to capture the behavior of the underlying model due to the structure of its mean and kernel functions.

There are several issues that should be addressed to perform efficient modeling:

- Basic approach allows modeling only a single output or multiple independent outputs, whereas our aim is to capture both geo-spatial and inter-feature dependencies at once.
- Naive GPR computational requirements increase cubically with the dataset size, as it requires matrix inversion.
- GPR model requires selection of multiple hyper-parameters, e.g., kernel and mean functions.

#### Multi-task Gaussian process regression

Let’s consider a more complex model than in the Eq. (2):

4 $$\begin{aligned} \mathbf {y}(\mathbf {x}) = {\mathbf {f}}(\mathbf {x}) + \epsilon , \end{aligned}$$

where $\mathbf {y}$ is a vector of *M* measured properties, $\epsilon \sim {\mathscr {N}}(0, D)$ with *D* being an $M \times M$ diagonal noise matrix. In order to capture both inter-feature and geo-spatial dependencies in covariance function construction, we refer to multi-task approach^59^:

5 $$\begin{aligned} k_{kl}(\mathbf {x}, \mathbf {x}^\prime ) = \langle f_k(\mathbf {x}), f_l(\mathbf {x}^\prime )\rangle = K^f_{kl} k^x(\mathbf {x},\mathbf {x}^\prime ),\, k,l=1,\dots ,M \end{aligned}$$

where $K^f$ is an $M \times M$ inter-feature covariance matrix and $\langle \cdot ,\cdot \rangle$ denotes a scalar product. Then, given the training data $\mathbf {X}=\left( \mathbf {x}_1,\dots ,\mathbf {x}_N\right) ^\intercal \in {\mathbb {R}}^{N \times d}$, ${\mathbf {Y}}=\left( \mathbf {y}_1,\dots ,\mathbf {y}_N\right) ^\intercal \in {\mathbb {R}}^{N\times M}$, the predictive distribution at the unobserved point $\mathbf {x}_*$ is found as

6 $$\begin{aligned}&{\hat{f}}(\mathbf {x}_*) \sim {\mathscr {N}}({\hat{\mu }}, {\hat{\Sigma }}),\\ {\hat{\mu }}(\mathbf {x}_*)&= \mu (\mathbf {x}_*) + (K^f \otimes k_*^x)^T\Sigma ^{-1}({\overline{\mathbf {y}}} - {\overline{\mu }}(\mathbf {X})), \\ {\hat{\Sigma }}(\mathbf {x}_*)&= K^f k^x(\mathbf {x}_*, \mathbf {x}_*) - (K^f \otimes k_*^x)^T\Sigma ^{-1} (K^f \otimes k_*^x), \\ \Sigma&= K^f \otimes K^x + D \otimes I, \end{aligned}$$

where $\otimes$ denotes a Kronecker product, ${\overline{\mathbf {y}}}$ and ${\overline{\mu }}$ are flattened $N \cdot M$-dimensional vectors obtained from $N\times M$ matrices $\mathbf {y}$ and $\mu (\mathbf {X})$, respectively. As a “side-effect” of this approach, after the model is built and hyper-parameters are optimized, we can analyze the dependencies between modeled properties using matrix $K^f$.

#### Decreasing computational complexity

One of the main disadvantages of the proposed multi-task GPR approach is that it induces a lot of additional calculations. In the naive case it becomes $O(N^3M^3)$ instead of $O(N^3)$, and to alleviate it, we turn to GPU computations. We perform modeling using Python programming language, GPyTorch library^60^ based on the PyTorch framework^61^ and NVIDIA Tesla K80 GPU. However, to further improve performance and to easily account for the possibly correlated components, we parametrize covariance matrix $K^f$ using low-rank approximation as follows:

7 $$\begin{aligned} K^f = B^TB + \mathrm {diag}({\mathbf {v}}), \end{aligned}$$

where *B* is an $M \times r$ matrix, ${\mathbf {v}}$ is an *M*-dimensional positive vector and *r* is the supposed rank of $K^f$.

#### Hyper-parameter selection

In order to simplify the hyper-parameter selection, we refer to *Spectral Mixture Kernel*^62^, which can be represented as:

8 $$\begin{aligned} k^x(\mathbf {x}, \mathbf {x}^\prime ) = k^x(\mathbf {x}-\mathbf {x}^\prime ) = k^x(\tau ) = \sum _{q=1}^{Q} w_{q} \prod _{p=1}^{d} \exp \{-2\pi ^2\tau _p^2v_p^{(q)}\}\cos \left( 2\pi \tau _p\mu _p^{(q)}\right) , \end{aligned}$$

where *Q* is the number of components in the mixture, $w_q$ is a weight of the $q^\text {th}$ component, $v_p^{(q)}$ and $\mu _p^{(q)}$ are $p^\text {th}$ variance and mean of the $q^\text {th}$ mixture component, respectively. The weights influence the importance of each separate component in the mixture, whereas variance and the mean allow to model effects of different scales. Hence, we are able to model arbitrary stationary kernels and control the complexity with a number of components *Q* in the mixture. Depending on the size and structure of the input data, the number of model parameters may require certain tuning. The main advantage of this approach is that it does not require any handcrafting of the potentially effective kernels, but instead, enables automatic hyper-parameter selection and optimization. Since the kernel assumes stationarity, we use a quadratic polynomial in two variables as the mean function to eliminate potential trend in data:

9 $$\begin{aligned} \mu (\mathbf {x}) = {\mathbf {c}}_0 + {\mathbf {c}}_1 \cdot x_1 + {\mathbf {c}}_2 \cdot x_2 + {\mathbf {c}}_{12} \cdot x_1 x_2 + {\mathbf {c}}_{11} \cdot x_1^2 + {\mathbf {c}}_{22} \cdot x_2^2 \end{aligned}$$

where ${\mathbf {c}}_0,\,{\mathbf {c}}_1,\,{\mathbf {c}}_2, {\mathbf {c}}_{12}, {\mathbf {c}}_{11}, {\mathbf {c}}_{22}$ are vectors of size *M* also being optimized during the model training. Optimization of the hyper-parameters is performed with MLE approach by solving the following maximization problem numerically:

10 $$\begin{aligned} \max _{\mathbf {\theta }}~\log p({\mathbf {Y}} | \mathbf {X}, \mathbf {\theta }) = -\frac{1}{2}({\overline{\mathbf {y}}} - {\overline{\mu }}(\mathbf {X}))^T \Sigma ^{-1}({\overline{\mathbf {y}}} - {\overline{\mu }}(\mathbf {X})) - \frac{1}{2}\log |\Sigma | - \frac{NM}{2}\log {2\pi }, \end{aligned}$$

where $\mathbf {\theta }$ denotes all hyper-parameters of the model.

### Data normalization

Spatial coordinates first were converted from EPSG:4326 (latitude, longitude) format to EPSG:32637 (UTM zone 37N) and, then, scaled down to [0, 1] range using the min-max normalization.

The scaling of the measured properties required a more complex approach. All of the properties are limited from below by zero and from above with some reasonable values, e.g., concentration can not be more than 100%, and, as another example, pH values can lie only in [0, 14] range. Unfortunately, a direct GPR does not consider such limits on outputs as the predictive distribution is normal and has infinite support. To incorporate such bounds we follow a *warping* approach^63,64^, which allows to map measurements from bounded space to unbounded and apply GPR directly. First, let *M*-size vectors ${\mathbf {b}}^L$ and ${\mathbf {b}}^U$ denote bounds of parameters dictated by regulatory documents from the Table 1. For every modeled parameter without the explicit value domain (all, except for pH in our case) we calculate their maximum values across all of the measurements ${\mathbf {y}}^{max} = \max \limits _{1\le i\le N} {\mathbf {y}}_i$. Then, we choose the maximum between the obtained values and ${\mathbf {b}}^U$ and multiply it by 10 (to certainly avoid out-of-bounds problem), thus, defining upper limits as $10 \cdot \max \{\mathbf {y}^{max}, {\mathbf {b}}^U\}$. The boundaries for pH are set as [0, 14] and the obtained limits are used for the min-max scaling, mapping all of the parameters to [0, 1] region. Furthermore, to map the scaled parameters to an unbounded space we use the inverse cumulative-distribution function (ICDF) of the standard normal distribution. Unfortunately, it can not be applied straightforwardly, because our dataset contains strict zero values (which coincide with lower bounds), thus, yielding $-\infty$ values after the transformation. To tackle this issue, we simply replace zeros with sufficiently small values of $10^{-10}$ before applying ICDF. Finally, we use another min-max scaling to end up with [0, 1] range of values. Thus, GPR predictive mean ends up in the required bounds after the appropriate inverse steps. Noteworthily, predictive distribution is Gaussian in the transformed measurement space, however, it is different in the original space and heavily depends on the warping function.

### Validation

To validate and compare the trained models we apply a standard cross-validation scheme with 5 random splits, with 80% and 20% of a train and test data, respectively. For each split we (i) perform the model fitting on training data, (ii) obtain predictions for each modeled property for the test data points and (iii) calculate $R^2$-score (or the coefficient of determination). This quality metric shows the proportion of the observed variation explained by the variation in the input data using the model. It is equal to one (1) if a predictive error is zero (0), zero (0) if a predictive error equals the test data variance, and negative if it is larger. The particular choice of the metric based on the Mean Square Error (MSE) is justified by our model selection. The predictive mean of GPR is tightly connected with a solution of Kernel Ridge Regression^65^, which involves the minimization of the exact MSE of the training data with additional regularization. As the model is intrinsically trained to minimize the Euclidean error, it is natural to use the MSE-based metrics for its evaluation. To select the best model we have to disregard the poorly modeled properties. Thus, we average $R^2$-scores over all splits and remove all of the properties for which the maximum, calculated over every model, yielded a negative value. Then, we average the scores over all kept properties and splits and select the model with the highest value.

### Probabilistic substance quality index

To evaluate the water quality we propose a new technique that takes advantage of GPR and allows to incorporate regulatory standards into assessment procedure directly. First, we note that the output of the GPR model is not just a vector of predicted values of properties, but a probabilistic distribution. Namely, at any location it gives a multi-dimensional normal distribution $\mathbf {z}\sim {\hat{p}}(\mathbf {z}~|~ \mathbf {x}_*) = {\mathscr {N}}\left( {\hat{\mu }}(\mathbf {x}_*), {\hat{\Sigma }}(\mathbf {x}_*)\right)$ described by the mean vector and covariance matrix from the group of Eqs. (6). Second, we appeal to the fact that the water quality is considered high if concentrations of different elements are located in admissible safe bounds, defined by governmental standards. Taking into account the above mentioned, we propose a measure coined Probabilistic Substance Quality Index (PSQI), which depicts the probability that all the measured properties will be within the admissible bounds. Therefore, it seems natural to integrate the probability density function ${\hat{p}}(\mathbf {z}~|~ \mathbf {x}_*)$ over these bounds. Unfortunately, due to the “curse of dimensionality”, an increase in the number of properties leads to a drastic decrease of the integral value and overall interpretability. Thus, we utilize the marginalization approach instead and define PSQI as follows:

11 $$\begin{aligned} \begin{aligned} \mathrm {PSQI}(\mathbf {x}_*)&= \sum _{i=1}^M w_i \cdot {\hat{p}}_i(\mathbf {x}_*), ~\sum _{i=1}^M w_i = 1, \\ {\hat{p}}_i(\mathbf {x}_*)&= \int _{-\infty }^{+\infty } \dots \int _{b_i^L}^{b_i^U} \dots \int _{-\infty }^{+\infty } {\hat{p}}(\mathbf {z}~|~\mathbf {x}_*) \mathrm {dz}_1\dots \mathrm {dz}_M, \end{aligned} \end{aligned}$$

where $w_i$ denotes the importance of each individual property in water quality and ${\mathbf {b}}^L$, ${\mathbf {b}}^U$ are admissible bounds for parameters from Table 1. By its construction, it is normalized to [0,1] interval, where zero value corresponds to zero possibility that properties are within the admissible bounds (bad quality) and one corresponds to the opposite (good quality). Since the integral in Eq. (11) does not have an analytical solution for the arbitrary bounds, we use *SciPy* library^66^ to perform numerical integration of multivariate Gaussian probability density.

Unfortunately, PSQI alone does not allow us to distinguish between the following cases of the index values being small: (a) predictive mean lies outside of the bounds and predictive variance is small (i.e., water is bad and we are certain about it); and (b) predictive mean lies inside of the bounds and predictive variance is large (i.e., water is good, but we are uncertain about it). To tackle this issue, we propose an additional confidence metric:

12 $$\begin{aligned} \begin{aligned} \mathrm {conf}(\mathbf {x}_*)&= \sum _{i=1}^M w_i \cdot {\hat{q}}_i(\mathbf {x}_*), ~\sum _{i=1}^M w_i = 1, \\ {\hat{q}}_i(\mathbf {x}_*)&= \int _{-\infty }^{+\infty } \dots \int _{b_i^L}^{b_i^U} \dots \int _{-\infty }^{+\infty } {\hat{q}}(\mathbf {z}~|~\mathbf {x}_*) \mathrm {dz}_1\dots \mathrm {dz}_M, \\ {\hat{q}}(\mathbf {z}~|~ \mathbf {x}_*)&= {\mathscr {N}}\left( ({\mathbf {b}}^L + {\mathbf {b}}^U)/2, {\hat{\Sigma }}(\mathbf {x}_*)\right) . \end{aligned} \end{aligned}$$

**Figure 3:**
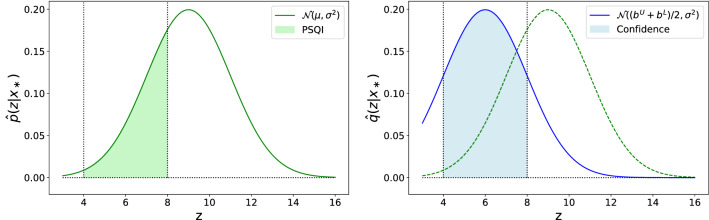
Example of PSQI (left) and confidence (right) calculation procedures for a single parameter. Dotted vertical lines denote admissible bounds $b^L$ and $b^U$, green and blue solid lines denote obtained predictive distribution and its centered variant, respectively. Both PSQI and confidence values are calculated as the area under the respective distribution curves between the bounds.

Confidence is calculated similarly to PSQI, although with an important difference—predictive distribution is centered within the bounds. This way, the confidence value is high, if the standard deviation is much smaller than the bounds and low, otherwise. One can note that PSQI and confidence values are interconnected, e.g., if the predictive distribution is already centered within admissible bounds, then, they match. Figure 3 shows PSQI and confidence calculations for a single modeled parameter ($M=1$).

The selection of weights $w_i$ can be governed by the hazardousness of deficiency or excess of individual properties. Unfortunately, it may be not very straightforward to choose particular values of the weights with such an approach. Instead, we use the model training results to determine the “importance” of model properties for PSQI computation. During training, we apply five-fold cross-validation and select the best model based on average $R^2$-scores for each modeled property. It is worth noting that $R^2$-score computed in transformed and original measurement space is different due to the non-linear nature of transformation (see in “Data normalization” section). Thus, we perform the model selection based on $R^2$ computed in the original state space. For some of the properties the model can perform poorly and yield negative $R^2$-score values, which implies that the simple mean has a better predicting capacity than the model. In this case, the properties with non-positive $R^2$-scores of their predictive means and variances are replaced with respective dataset means and variances, and all their inter-parameter correlations are considered zero. Further, to deal with the discrepancy of prediction accuracy among different properties, we propose to compute weights from Eqs. (11) and (12) using $R^2$-scores and *softmax* function:

13 $$\begin{aligned} w_i = \frac{\exp \left( \max (R^2_i, 0)\right) }{\sum _{i=1}^M \exp \left( \max (R^2_i, 0)\right) }, \end{aligned}$$

where $R^2_i$ denotes $R^2$-score obtained for $i^\text {th}$ property during training. This way, we incorporate modeling accuracy into PSQI computations directly and reduce potential over- or under-estimations of the index. Moreover, it is still possible to incorporate ad-hoc importance of particular properties with an additional re-weighting.

## Results

The source code, data, and results can be found in our repository^67^ with available interactive visualization via kepler.gl platform.

### Experiment

To apply and analyze the proposed approach we used a dataset obtained from a large environmental investigation in the New Moscow area, Russia. Firstly, we modeled spatial distribution of multiple available parameters, Alkalinity, Hardness, Mineralization, pH, ions of sodium (Na), potassium (K), calcium (Ca), magnesium (Mg), manganese (Mn), iron (Fe), copper (Cu), nickel (Ni), chrome (Cr), zinc (Zn), bicarbonate (HCO$_3$), ammonium (NH$_4$), nitrate (NO$_2$), nitrite (NO$_3$), chloride (Cl), orthophosphate (PO$_4$), and sulfate (SO$_4$), implementing multi-task GPR framework (see in “Gaussian process regression” section).

**Figure 4:**
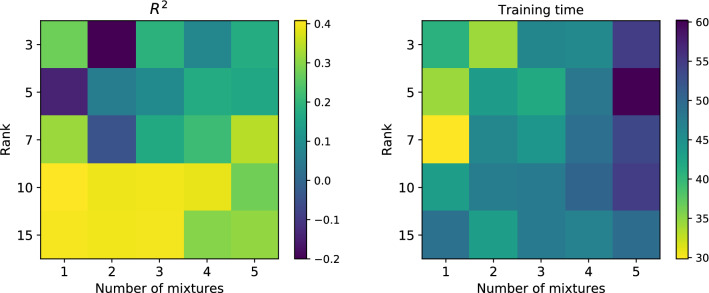
$R^2$-score averaged over every split and well-modeled property (left) and complete training time of a model in seconds (right) for different numbers of mixture components *Q* and ranks *r* of the inter-feature covariance matrix $K^f$. Increase in number of mixtures leads to over-parametrization and degradation in both accuracy and computational speed. Whereas, increase of the rank improves accuracy with a reasonable increase of computational time.

To avoid serious over-fitting during the training phase, we bounded the corresponding length-scales of the mixture components within [0.1, 100] interval. We trained several models with different number of mixtures *Q* (from 1 to 5) in the spatial kernel (see Eq. 8) and different ranks *r* (3, 5, 7, 10, 15) of the inter-feature covariance matrix $K^f$ (see Eq. 7). To select the best model, we considered prediction accuracy only for 20 components, except for Cr as it yielded very poor results for every model. Figure 4 shows the average $R^2$-score over every kept component and split for each model for different values of *Q* and *r*. It can be seen, that increasing model complexity does not necessarily lead to model improvements, as it can bring about over-fitting. Moreover, it typically causes time-performance degradation. The best model corresponds to a single mixture component and rank-10 covariance matrix. Training of the best model took 6.8 s, whereas evaluation over test data took 0.6 s.

### Modeling

**Figure 5:**
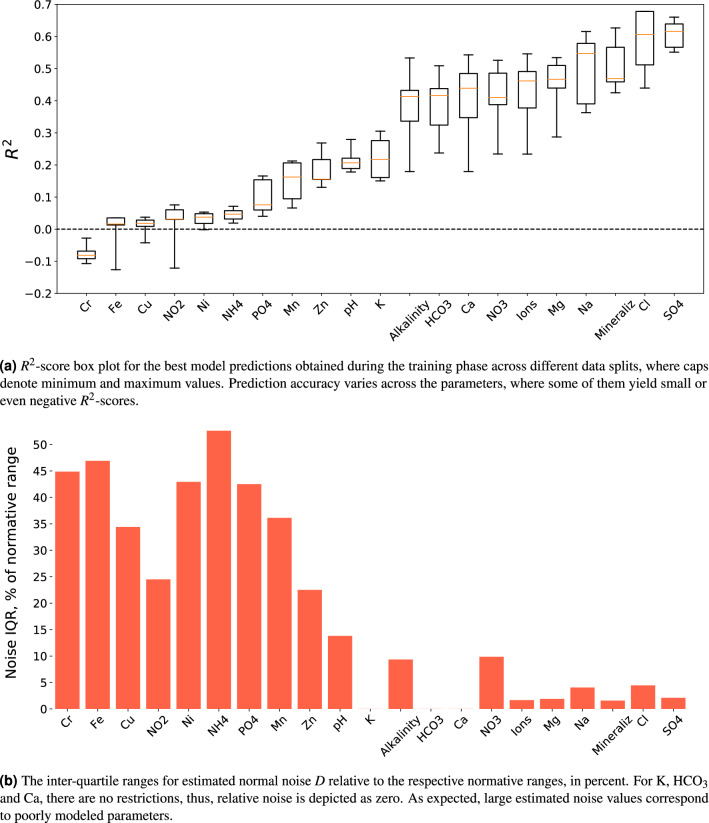
Modeling results.

Figure 5a illustrates per-parameter performance with average, min, and max values of $R^2$-score over splits for different properties obtained using the best selected model. We can see that some of the properties were predicted very poorly, such as Cr, Fe, Ni, Cu, NH$_4$, NO$_2$ with the best average score of 0.635 for SO$_4$. One of the reasons for the low accuracy may be a high level of noise in the data. It is accounted in the model and estimated during the training phase as an additive Gaussian component with the covariance matrix *D* from Eq. (4). Large noise values tend to correspond to a poor fit of the model given the training data for a particular water property. Unfortunately, it is not possible to illustrate the noise values in the original state space straightforwardly. The reason is that the modeling is done in normalized parameter state space (see in “Data normalization” section), therefore, in the original space normal noise is not obliged to be normal anymore. We illustrate it with Fig. 5b in a more comprehensive way, dividing the inter-quartile range for each noise component by the respective normalized normative range. For water properties without established restrictions, i.e., K, HCO$_3$, Ca, relative noise is zero, and for poorly modeled properties relative noise is very high.

**Figure 6:**
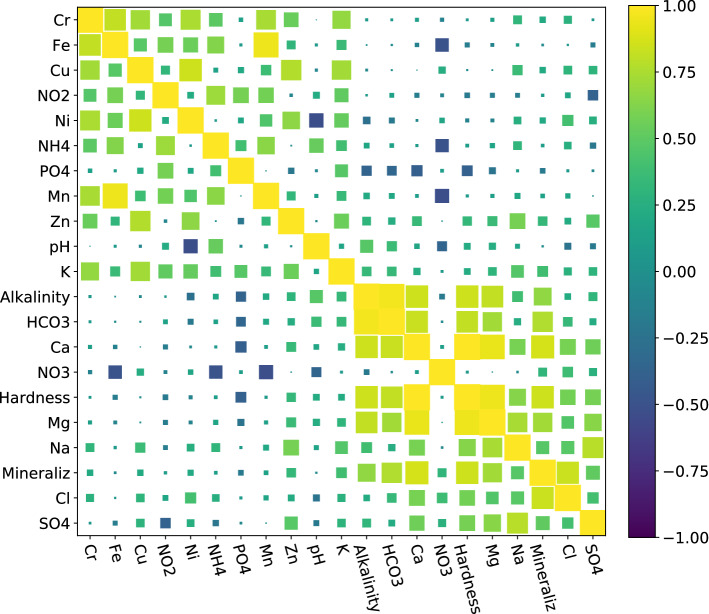
Pearson correlation matrix for the modeled properties derived from the optimized $K^f$ covariance matrix. Yellow (dark blue) color denotes positive (negative) correlation, whereas wide (narrow) boxes represent strong (weak) correlation.

As a “side-effect” of the model construction we obtained the optimized $K^f$ covariance matrix describing interconnections of water properties, for which, corresponding correlation matrix is shown in Fig. 6. General characteristics such as Alkalinity, Hardness and Mineralization rates are highly correlated with HCO$_3$, Ca, Mg, correlation coefficients ($\rho$) lay in diapason from 0.63 to 0.99, while the presence of SO$_4$ mostly correlates with ions of Na, Mg, correlations are 0.79 and 0.65, respectively. Apart from that, highest correlations (more than 0.6) are observed in pairs: $\rho$(Cr & Fe) = 0.81, $\rho$(Cr & Cu) = 0.73, $\rho$(Cr & Ni) = 0.75, $\rho$(Cr & Mn) = 0.74, $\rho$(Cr & K) = 0.68, $\rho$(NH$_4$ & Fe) = 0.63, $\rho$(Cu & Ni) = 0.85, $\rho$(Cu & Zn) = 0.77.

### PSQI

**Figure 7:**
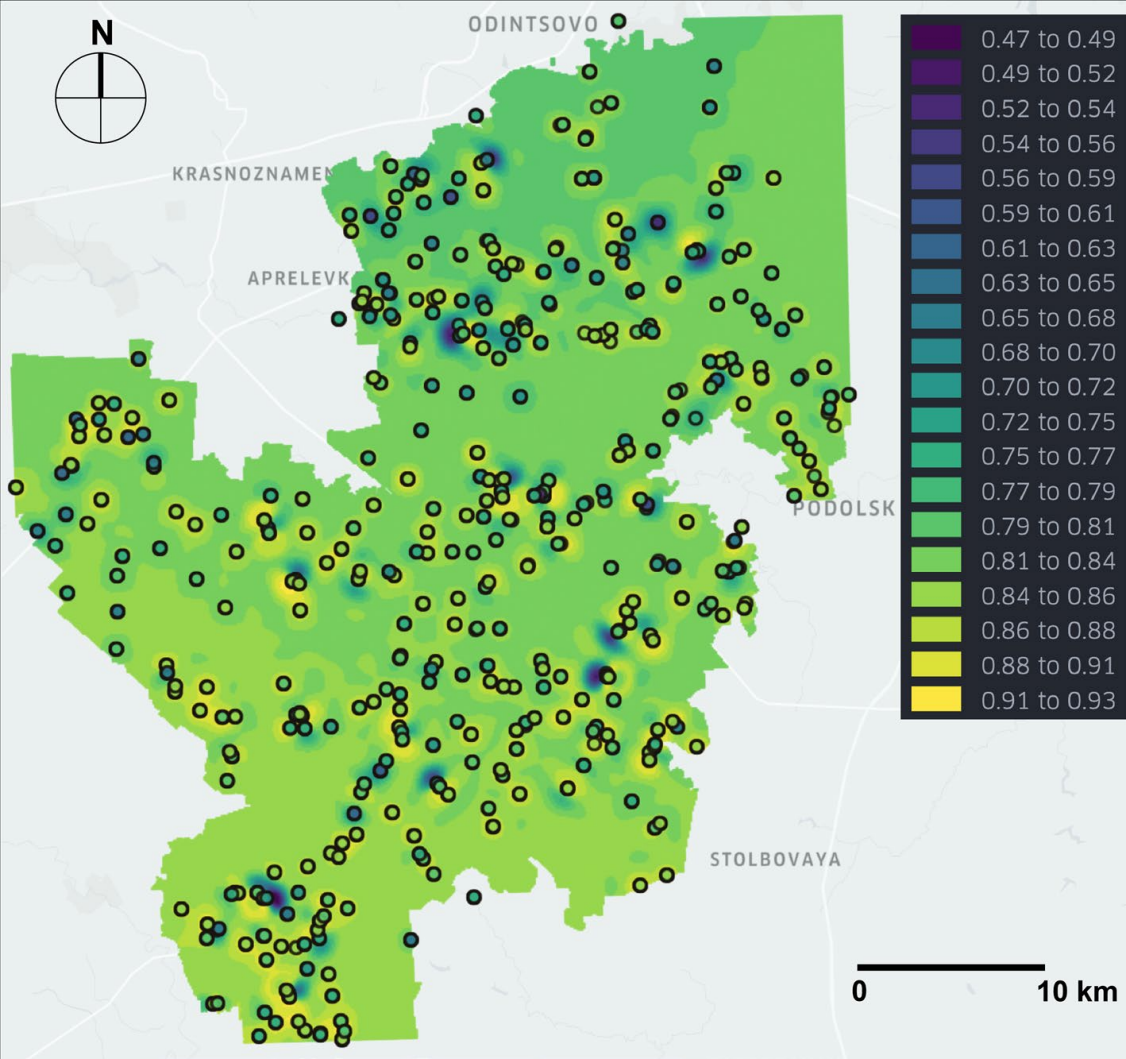
Geo-spatial map of predicted PSQI values. Dark violet color represents low PSQI values, whereas light yellow - high PSQI values. Outlined points are sampled measurements with color representing the fraction of properties that appear to be in admissible bounds. The map was created with Kepler.gl platform (v2.5.1).

In order to calculate the PSQI values, we have used admissible bounds reflected in local regulations of the Russian Federation (see Table 1). Some of the parameters do not have any regulatory restrictions (Ca, K and HCO$_3$), thus, we excluded them from the calculation of PSQI to avoid overestimation. As could be noted earlier from Fig. 5, the prediction quality of our model differs across the properties and for some of them even yield negative $R^2$-scores. Calculation of PSQI at a single point comprises two steps: (a) evaluation of the predictive distribution; (b) computations from Eqs. (11) and (12) (see in “Probabilistic substance quality index” section). We chose the best performing model during the validation stage, fixed all of its hyper-parameters, and used the whole dataset to make predictions at locations of interest. They were uniformly selected across the New Moscow region with 100 $m^2$ resolution, giving 151 447 points. The evaluation of the predictive distribution took 5.8 hours with approximately 130 ms per point. The subsequent computation of PSQI took only 95 seconds, which can be considered negligible. Figure 7 shows the spatial distribution of PSQI values obtained from predicted distributions, where outlined points denote collected samples. To additionally validate that PSQI indeed corresponds to the fraction of measured parameters being in admissible bounds, we, (i) evaluated PSQI at each sampling location, (ii) calculated such fraction directly for each measurement, and finally (iii) computed Pearson correlation coefficient between them, resulting in the reasonably large value of 0.68. The spatial distribution of PSQI confidence appeared to be of no practical interest, due to its very small scatter from 0.935 to 0.956 with 55% points yielding values less than 0.936.

## Discussion

A typical modeling task consists of several pre-processing steps, one of which may be dimensionality reduction used to disregard the non-informative features or find the most “independent” combinations of the features to build the model for. It may help to decrease the computational complexity, but requires a careful dataset pre-processing and leads to information losses. Multi-task GPR allows to perform simultaneous geospatial modeling and capture the inter-feature dependencies while being able to control complexity using parametrization techniques. However, the computational overhead may reach as much as $O(N^3M^3)$, thus, requiring further considerations of performance improvements^50,51,68–71^.

One of key issues for geo-spatial modeling is the model construction process itself. On the one hand, manual selection of the kernel function based on domain knowledge allows to adapt to different areas of application. On the other hand, it limits automatization and scalability of the modeling significantly and causes some difficulty for integrating it into support-decision systems. Spectral Mixture Kernel facilitates the task of model construction, giving the ability to approximate arbitrary stationary kernels and control the accuracy with the number of mixtures. Since the number of mixtures is discrete, it can not be effectively optimized using MLE. In this case multiple models (for the different number of mixtures) can be trained and compared using the Cross-Validation technique to pick the best overall solution. However, the increase in the model complexity may lead not only to accuracy improvements, but on the contrary to the over-parametrization and degradation of both computational speed and the quality of predictions. Therefore, effective model construction still requires thorough consideration of both domain knowledge and the dataset structure.

Freshwater characteristics usually depend on various factors such as the intensity of geological and hydrogeological settings due to dissolution processes and ion exchanges; seasonal fluctuations and climate change in global, being also affected by anthropogenic loads^72^. The modeled properties show reasonable correlations (Fig. 6), adequate for the natural freshwater resources and explainable for those influenced by urbanization and agricultural activity widespread across the territory of sample net. Ions of HCO$_3$, Ca, Mg, SO$_4$, Na, Mg, being major macro constituents, are always presented in groundwater, their concentrations depend mostly on the mineral composition of rocks, although surrounding lands as well^73^. The presence of nitrogen forms in the ground and surface water can accompany the agricultural and landfill sites, relating to the migration of products of fertilizers, pesticides or domestic sewage decay^74–77^. The observed correlations between NH$_4$, NO$_2$, NO$_3$ are ranged according to the stages of oxidation transformation of NH$_4$. Additionally, substantial correlations between Cr, Fe, Cu, Ni, Mn, K, as well as between PO$_4$ and K also often are explained by the migration of fertilizers’ degradation residuals across the landscape and into water sources. Apart from that, high intercorrelation between trace elements in the water samples can be linked to the objects with a high pollution potential, such as landfills, transport systems, industry. Among them, Ni demonstrates a remarkable negative correlation of -0.52 with pH, which can be explained by the reduced migration ability in alkaline conditions and can be supported by the noticeable correlation of -0.48 between pH and Alkalinity.

Although more sophisticated variations of WQI have been recently proposed (e.g. based on multi-criteria decision analysis (MCDA)^28^, entropy-weighted indices^30^, modified by principal component analysis for optimized parameter selection^29^), most of the existing WQI solutions have limitations, e.g. low independence of expertise and, as a consequence, site and case specificity as well as low robustness. In general, estimation of WQI includes the following steps: scaling of the selected parameters if they have different dimensions; selection of the most important parameters according to some rule, including an a priori knowledge; determining the relative weight of each parameter; calculating the sub-index of each parameter from the relative weight, and, finally, summarizing the results and determining the quality rating scale^78–80^. However, subjective judgments may cause certain confusion, e.g., in the determination of parameters importance, in the choice of the weight values for each parameter, in comparing the sum with expert-opinion-based ranges, as well as different limits scales of parameters.

As compared to the methodologies discussed above the proposed PSQI is directly linked to the established water quality guideline standards for each characteristic itself. PSQI does not rely on the subjective judgments about parameters’ importance neither on the structure of the input data. Additionally, PSQI covers admissible limits not only as single values, e.g., when properties must be lower than specific upper bound, but allows to consider an optimal range, such as for pH or alkalinity. This is an important improvement of the currently used techniques and it makes the proposed solution applicable for the assessment of other environmental media, e.g. in the case of soils favorable content of macro and micro-nutrients or physical properties are expressed as optimum ranges^81,82^.

## Materials

### Study area

The data was collected in the 2017–2018 years. A detailed description of the territory and the sample net is provided in Shadrin et al^83^. In a nutshell, the sample net is mostly located across the New Moscow region, Russia. According to the official statistics and research reports New Moscow is characterized by the rapid rise of both urban areas and density during the last 10 years^84,85^. The New Moscow is located close to the bottom boundary of southern taiga, in the Central European part of Russia (55$^{\circ }$N, 37$^{\circ }$E) and extends over 1480 km $^{2}$ in area. The mean annual temperature of this region is about 3-4$^{\circ }$ C. The territory includes all of the common types of land-uses, including urban fabric, forests, and green urban areas, arable lands, industrial cites. The predominant types of natural vegetation are coniferous and broad-leaved forests, while agricultural lands include pastures and arable land mostly growing feed crops and cereals^86^. The mean temperature in the coldest month of the year (January) ranges between -9.5$^{\circ }$ C and -11.5$^{\circ }$ C, while in the warmest month, July, mean temperatures are between +17$^{\circ }$ C and +18.5$^{\circ }$ C. The average annual precipitation is approximately 400-500 mm, with around two-thirds by rainfall and the rest by snow, according to recent observations from weather stations’ net across the territory (available at https://www.ncdc.noaa.gov/cdo-web/datatools/findstation). The territory has a plain topographic relief, and the bedrock consists of glacial and fluvioglacial loams and sands with the inclusion of sandy alluvial deposits.

### Dataset description

The analytical samples were collected from different sources of freshwater, namely: wells, rivers, and springs from the territories of private households. Some of the sample points included the replicated measurements, which have been taken into account at both modeling and results’ interpretation stages. Overall, the dataset includes 1569 samples at 460 unique points, each sample consists of longitude, latitude, and list of chemical compounds content and properties, commonly used for water quality assessment. The normative ranges for measured properties are given according to the Russian regulation documents—SanPiN (Sanitary Rules and Norms), number 1.2.3685-21 being in force at the time of the manuscript preparation. These values were given as an example for study support and can be changed according to any other guideline source. Although being measured, Hg, Cd, Co, Pb were eliminated from further work as having very low variability across the dataset. The data points sampled too far from the main research area were removed. Finally, for further modeling, we used 1526 data vectors of 21 properties, namely Alkalinity, Ca, Cl, Cr, Cu, Fe, HCO$_3$, Hardness, K, Mg, Mineralization, Mn, NH$_4$, NO$_2$, NO$_3$, Na, Ni, PO$_4$ SO$_4$, Zn, pH (see Table 1).

**Table 1:** List of parameters of study dataset and their basic statistics: minimum (**Min.**), first quartille (**1st Qu.**), median, mean, third quartile (**3rd Qu.**), maximum (**Max.**) and normative range, represented according to sanitary regulations in Russia.

Parameter	Dimension	Min.	1st Qu.	Median	Mean	3rd Qu.	Max.	Normative range	
	pH	–	5.50	6.73	7.10	7.04	7.40	8.40	6–9
Alkalinity	mg-eq/L	0.50	3.45	4.50	4.68	5.84	12.00	0.5-6.5	
Hardness	mg-eq/L	0.60	4.20	5.60	5.74	6.90	21.90	7	
Mineraliz	mg-eq/L	37.00	281.80	366.00	402.60	481.00	1586.00	1000	
Ca	mg/L	8.85	63.60	82.17	85.74	101.90	340.00	–	
Mg	mg/L	1.48	12.01	16.81	17.67	22.04	60.44	50	
Na	mg/L	0.00	9.99	16.19	26.22	31.22	245.00	200	
K	mg/L	0.00	1.09	2.59	8.56	6.94	181.80	–	
NH$_4$	mg/L	0.00	0.00	0.06	0.54	0.40	38.00	2	
HCO$_3$,	mg/L	31.00	210.00	275.00	285.70	356.20	732.00	–	
Cl	mg/L	0.00	12.26	26.07	53.96	59.29	748.41	350	
NO$_3$	mg/L	0.00	5.18	17.16	27.20	37.61	352.81	45	
NO$_2$	mg/L	0.00	0.00	0.00	0.02	0.00	2.25	3	
PO$_4$	mg/L	0.00	0.00	0.00	0.35	0.00	15.32	3.5	
SO$_4$	mg/L	0.80	20.53	34.08	40.44	52.20	246.14	500	
Cr	mg/L	0.00	0.00	0.00	0.00	0.00	0.04	0.05	
Cu	mg/L	0.00	0.00	0.00	0.00	0.00	0.13	1	
Fe	mg/L	0.00	0.04	0.14	0.34	0.32	18.52	0.3–1	
Mn	mg/L	0.00	0.00	0.01	0.06	0.04	3.12	0.1	
Ni	mg/L	0.00	0.00	0.00	0.00	0.00	0.27	0.1	
Zn	mg/L	0.00	0.01	0.04	0.15	0.11	4.90	5	

## Data Availability

The source code, data, and results are provided in our publicly accessible repository^67^ with available interactive visualization via kepler.gl platform and step-by-step instructions.
